# Assessing the Inhibitory Potential of Kinase Inhibitors In Vitro: Major Pitfalls and Suggestions for Improving Comparability of Data Using CK1 Inhibitors as an Example

**DOI:** 10.3390/molecules26164898

**Published:** 2021-08-12

**Authors:** Aileen Roth, Adrian Gihring, Florian Göser, Christian Peifer, Uwe Knippschild, Joachim Bischof

**Affiliations:** 1 Department of General and Visceral Surgery, Surgery Center, Ulm University Medical Center, Albert-Einstein-Allee 23, 89081 Ulm, Germany; aileen.roth@uni-ulm.de (A.R.); adrian.gihring@uni-ulm.de (A.G.); florian.goeser@uni-ulm.de (F.G.); joachim.bischof@uniklinik-ulm.de (J.B.); 2 Institute of Pharmacy, Christian-Albrechts-University of Kiel, Gutenbergstraße 76, 24118 Kiel, Germany; cpeifer@pharmazie.uni-kiel.de

**Keywords:** protein kinase, small molecule inhibitor, IC_50_, inhibitor constant, K_i_, workflow, standardization, CK1

## Abstract

Phosphorylation events catalyzed by protein kinases represent one of the most prevalent as well as important regulatory posttranslational modifications, and dysregulation of protein kinases is associated with the pathogenesis of different diseases. Therefore, interest in developing potent small molecule kinase inhibitors has increased enormously within the last two decades. A critical step in the development of new inhibitors is cell-free in vitro testing with the intention to determine comparable parameters like the commonly used IC_50_ value. However, values described in the literature are often biased as experimental setups used for determination of kinase activity lack comparability due to different readout parameters, insufficient normalization or the sheer number of experimental approaches. Here, we would like to hold a brief for highly sensitive, radioactive-based in vitro kinase assays especially suitable for kinases exhibiting autophosphorylation activity. Therefore, we demonstrate a systematic workflow for complementing and validating results from high-throughput screening as well as increasing the comparability of enzyme-specific inhibitor parameters for radiometric as well as non-radiometric assays. Using members of the CK1 family of serine/threonine-specific protein kinases and established CK1-specific inhibitors as examples, we clearly demonstrate the power of our proposed workflow, which has the potential to support the generation of more comparable data for biological characterization of kinase inhibitors.

## 1. Introduction

Posttranslational modifications of proteins are manifold and can be linked to various functional consequences for the mature polypeptide like the regulation of protein activity or stability. One of the most common posttranslational modifications is the phosphorylation of proteins. Protein kinases catalyze the reversible transfer of an ATP-derived γ-phosphate moiety to a substrate protein, either resulting in its activation, deactivation or even degradation [1,2]. Therefore, phosphorylation represents one of the most important regulatory mechanisms for cellular processes like protein synthesis, cell division or cell adhesion [3,4]. As a consequence, dysregulation of protein phosphorylation can be linked to the pathogenesis of various diseases, among others inflammatory, cardiovascular, and neurodegenerative diseases as well as cancer [5,6]. Within the past two decades, protein kinases attracted major attention as targets for inhibition mediated by small molecule inhibitors (SMIs). To date more than 100,000 SMIs for protein kinases have been described and characterized [7], from which 76 already have been approved by the local state authorities. However, in cancer research only 50 of the more than 500 human protein kinases have been exploited as drug targets so far [8].

A critical step in the development of new SMIs is the cell-free in vitro testing of novel inhibitor compounds with the intention to determine the IC_50_ values (concentration for 50% inhibition), specified by the amount of inhibitor required to reduce total enzyme activity by 50%. Although IC_50_ values as empirical values are generally established to compare the potency of SMIs, literature-derived values are often biased as kinase assays used for the determination of kinase inhibitory effects lack comparability due to varying experimental setups including different readout parameters or insufficient normalization. Thus, the use of IC_50_ values is only useful for quick comparison of various compounds tested in the same experimental setup, but it is inappropriate to compare data generated in different laboratories using different assay strategies. This lack of comparability can be overcome by using K_i_, an enzyme-specific variable that is independent of the experimental setup and universally comparable. Determining K_i_ can be difficult and never infallible, but extrapolating K_i_ from IC_50_ values of kinase inhibitors significantly contributes to comparability. As an example for the lack of comparability, IC_50_ values determined for established CK1-specific inhibitors are shown in Table 1.

In vitro kinase reactions are usually composed of recombinantly produced kinases used to phosphorylate either peptide or protein substrates. Not only the origin (e.g., human vs. yeast), the manufacturing process of the kinase protein (e.g., prokaryotic vs. eukaryotic expression systems, expression time, temperature during protein expression, purification via His vs. GST tag), or the presence of organic solvents (e.g., DMSO), but also the reaction conditions and readout technique selected to determine the parameters of the enzymatic reaction can be highly variable. In recent years, various label-free methods have been developed to determine kinase activity in vitro, including colorimetric, fluorimetric and mass spectrometry-based approaches, with readouts more approachable for high-throughput screenings as meanwhile also offered by numerous contract research organizations (CROs) [14]. Most of these approaches are designed to measure consumption of the co-substrate ATP or to determine the generation of ADP [15,16]. As different labeling strategies are used in high-throughput applications, an attenuation of accuracy and comparability has to be considered. Hence, the validation of high-throughput inhibitor data, comparable to the validation of mRNA microarray data by qRT-PCR results, seems to be indispensable in this field of research. This validation can be achieved by performing highly accurate, enzyme-optimized radiolabeling assays. In contrast to fluorescence- and luciferase-based kinase assays, phosphorylation of the substrate protein can be directly measured after providing radioactively labeled [γ-^32^P]-ATP or [γ-^33^P]-ATP as co-substrate to the kinase to be analyzed and by detecting incorporation of radioactively labeled phosphate into the substrate. Although working with radioactive substances is often considered as outdated and cumbersome, the high tractability and sensitivity of radiolabeled assays are among their major advantages. Further advantages are the use of any reaction solvents, natural substrates, a wide range of substrate concentrations, cell lysates, the possibility to identify specific phosphorylation sites and the direct validation of detected site-specific phosphorylation events by using phosphorylation-site substrate mutants. Lastly, the subsequent separation of the kinase reactions via SDS-PAGE also allows for discrimination between substrate phosphorylation and autophosphorylation of the kinase protein, which is essential in order to correctly and reliably determine kinase activity and thus inhibitor potency. Autophosphorylation modulates kinase activity and is a feature shared by almost all eukaryotic protein kinases [17,18], most of them becoming catalytically active by phosphorylation of their activation loop or autophosphorylation domains [19]. The levels of kinase autophosphorylation can be highly variable between kinases originating from different expression systems [20]. In case of auto-activating kinases different autophosphorylation levels can be overcome by preincubation with ATP prior to performing in vitro kinase assays with exogenous substrates. Unfortunately, a reliable pretreatment for auto-inactivating kinases, like CK1, does not exist and is limited to hardly reproducible procedures involving co-expression of or treatment with phosphatases [21]. Alternatively, autophosphorylation sites could be mutated or autophosphorylation domains truncated, however, resulting in artificially activated kinase proteins with disturbed sequence integrity [22,23]. Precisely for that reason autophosphorylation, disregarded by most of the commonly available screening methods, should be investigated prior to inhibitor testing and treated as an additional variable that can highly influence and distort results. Apart from the methods specifying the readout parameters for the assays, important basics of enzyme kinetics have to be taken into account when determining comparative values (e.g., IC_50_) for SMIs [24]. These include (i) the determination of the initial velocity region of the enzymatic reaction and (ii) the determination of the Michaelis constant (K_m_) and the inhibitor constant (K_i_) under the previously determined initial velocity conditions.

### 1.1. Determination of the Initial Velocity Region

**Figure 1:**
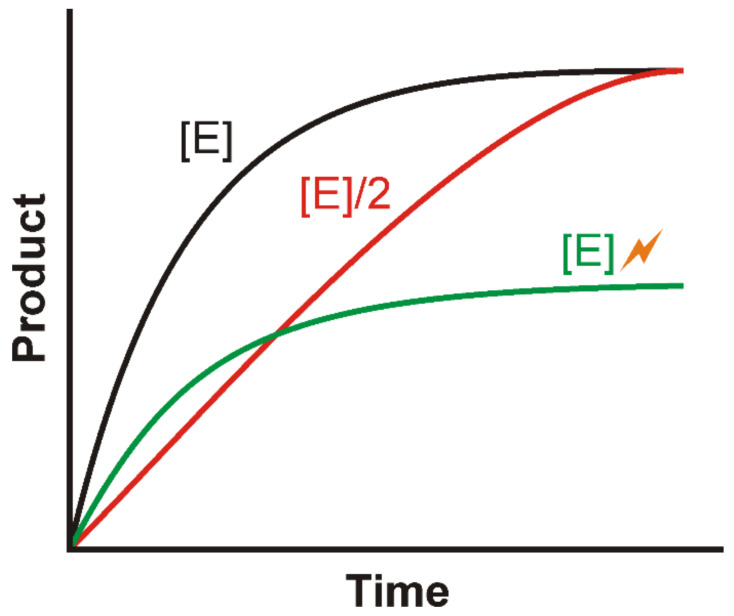
Exemplary product-over-time progression curves for an exemplary enzymatic reaction. The product-over-time curves are shown for different conditions including different enzyme concentrations ([E] and [E]/2 shown in black and red) and inhibited or degraded enzyme (green). [E], enzyme concentration.

For an enzymatic reaction, the initial velocity region describes the linear part of a product-over-time progression curve characterized by time-dependent increase in pro-duct concentration and a constant slope indicating the highest possible enzyme velocity [24]. With increasing time, the enzyme velocity (the slope of the progression curve) decreases due to a lack of substrate (Figure 1 black curve). By decreasing the enzyme concentration (red curve) the initial velocity region can be extended while simultaneously lowering the limit of detectable IC_50_ values. The shape of the initial velocity region can furthermore be affected by the loss of enzymatic activity due to inhibition (e.g., mediated by autophosphorylation [18], product inhibition [25]) or degradation (green curve). Determining the initial velocity region by testing different enzyme concentrations is a mandatory requirement for the correct subsequent determination of the kinetic parameters and should be considered as the first step of the inhibitor screening procedure. Ideal enzyme concentration and reaction time should be selected from the initial velocity region within a period in which no more than 10% of the total substrate concentration has been consumed [24]. By default, the defined enzyme concentration and reaction time should be used for the subsequent experiments.

### 1.2. Determination of K_m (ATP)_ and K_i_ under Initial Velocity Conditions

The potency of SMIs acting on various target enzymes is usually compared on the basis of their respective IC_50_ values. However, due to the use of different reaction setups, comparability of IC_50_ values can be limited and using the enzyme-specific K_i_ instead is considered to be advantageous. In order to calculate the K_i_ value, determination of the K_m_ for ATP has to be performed first. Therefore, the previously defined initial velocity conditions (enzyme concentration and reaction time) are used for testing different ATP concentrations ranging from 0.5 to 5 times K_m_ while maintaining the substrate to be phosphorylated under saturating conditions. Testing new enzymes with an unknown K_m_ value should follow an iterative process where the compliance of the ATP concentration range is subsequently evaluated and re-adjusted, if necessary. K_m (ATP)_ serves as an important reference value for the ATP concentration to be used in subsequent kinase assays for IC_50_ determination [26]. In order to obtain K_i_, we have to refer to the Cheng-Prusoff equation:

(1) $$K_{i}=\frac{{\mathrm{IC}}_{50}}{\left(\frac{\left[S\right]}{K_{m}}+1\right)}$$

For competitive inhibitors K_ic_ can easily be calculated if the used ATP concentration [S] in IC_50_ kinase reactions equals the K_m (ATP)_:

(2) $$\left[S\right]=K_{m}$$

In this case, Equation (2) can be substituted into Equation (1) and K_ic_ can be defined as half of the determined IC_50_ value:

(3) $$K_{\mathrm{ic}}=\frac{{\mathrm{IC}}_{50}}{2}$$

**Figure 2:**
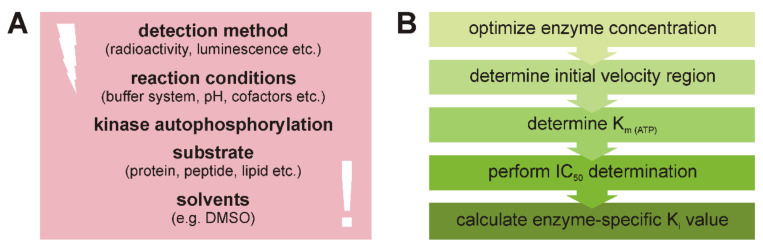
Considerations and suggested workflow for in vitro kinase inhibitor characterization. (**A**) Apart from basic considerations concerning the detection method to be used and the reaction conditions, also enzyme- and substrate-specific characteristics need to be taken into account. (**B**) The suggested workflow consists of five steps resulting in generation of major parameters essential to calculate K_i_ values, which can be easily used to compare inhibitory effects for various SMIs and different enzymes.

Consequently, using an ATP concentration equal to K_m (ATP)_ under initial velocity conditions allows for the direct conversion of the calculated IC_50_ into the enzyme-specific inhibitor constant K_ic_. Following the proposed workflow of determining the ideal enzymatic reaction parameters, and by implementing an experimental setup meeting various important requirements (summarized in Figure 2) it will be possible to determine enzyme-specific values for the reliable comparison of inhibitor potency on different enzymes. Using protein kinases of the CK1 family and several well-established CK1-specific SMIs as examples, we demonstrate the implementation and the significance of the proposed workflow. Although a radiometric determination approach is used in our demonstration, the workflow can easily be adapted to non-radiometric assays.

Members of the CK1 family and their respective splice variants are highly conserved serine/threonine protein kinases involved in the regulation of several fundamental cellular processes like stress response and cell cycle progression [27]. Due to their regulatory function in DNA damage-related signal transduction including the direct phosphorylation of central components like p53 and MDM2 as well as their involvement in Wnt, Hedgehog and Hippo signal transduction pathways CK1 isoforms have evolved as potential targets in cancer therapy [28,29]. Dysregulation and mutations of CK1 isoforms were shown to be involved in cancer progression but also play a critical role in the development of neurodegenerative diseases like Alzheimer’s disease [18,30]. According to this, the development of SMIs that exclusively inhibit disease-associated CK1 isoforms has been promoted in recent years.

## 2. Results

Demonstrating a workflow for the establishment of standardized and reproducible kinase reactions and kinase inhibitor characterization, the considerations and processes starting with the nature of recombinant kinase and finishing in the determination of IC_50_ or K_ic_ values are exemplarily demonstrated for the inhibition of protein kinase CK1δ. Simultaneously, the effects of different affinity tags, intramolecular autophosphorylation as well as organic solvents is being investigated. Finally, the universality and transferability of the proposed workflow as well as the superior comparability of K_ic_ instead of IC_50_ values is demonstrated by characterizing inhibitory effects of established inhibitors on another CK1 isoform.

### 2.1. Determination of Initial Velocity Region for GST-Tagged CK1δ

**Figure 3:**
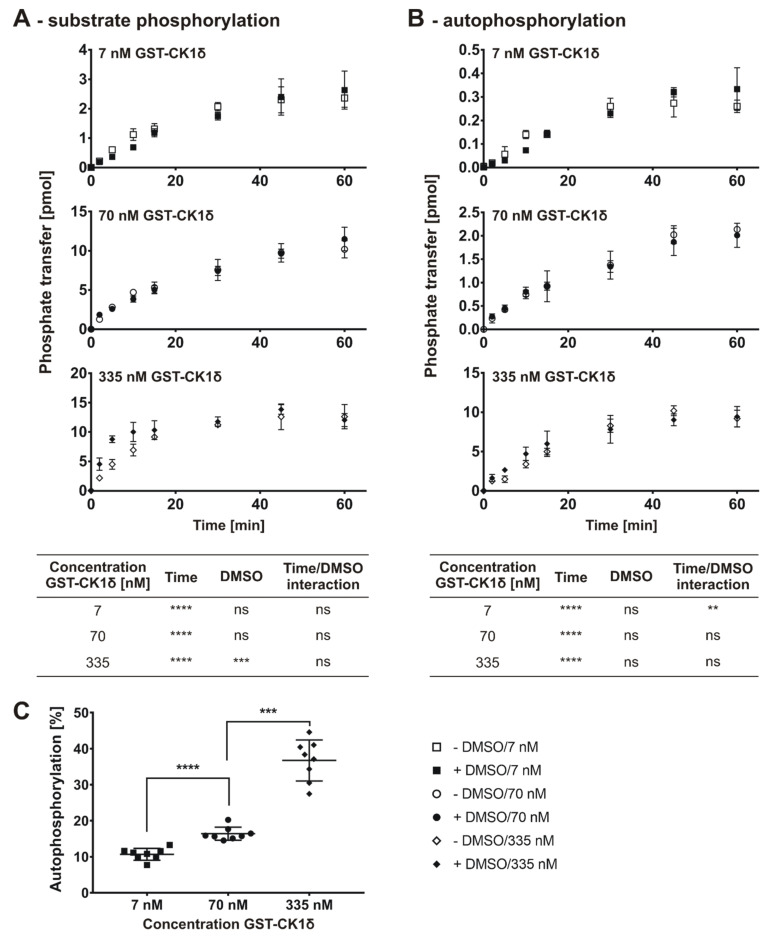
Product-over-time progression curve for GST-tagged CK1δ autophosphorylation and substrate phosphorylation. (**A**) Time-dependent phosphate transfer from [γ-^32^P]-ATP to the substrate α-casein catalyzed by human recombinant GST-tagged CK1δ using different enzyme concentrations (7, 70, 335 nM) was determined by in vitro kinase reactions in absence and presence of solvent (+/− DMSO). (**B**) Time-dependent autophosphorylation of GST-CK1δ has been determined and is presented similar to data shown in (**A**). (**C**) The mean proportional autophosphorylation relative to total phosphorylation (cumulated time-points) is significantly increased at higher enzyme concentrations. Statistical significance was tested using two-way ANOVA (for data presented in (**A**,**B**)) and unpaired two-tailed *t*-test (**C**). ns indicates *p* > 0.05, ** indicates *p* ≤ 0.01, *** indicates *p* ≤ 0.001, **** indicates *p* ≤ 0.0001. DMSO, dimethyl sulfoxide.

In a first step the initial velocity region for GST-tagged CK1δ was determined by measuring the time-dependent substrate phosphorylation of α-casein using different enzyme concentrations (7, 70, and 335 nM) (Figure 3A). For the determination of the initial velocity region, the phosphate donor ATP was used at a concentration of 10 µM (equal to 150 pmol per reaction). Additionally, the influence of the inhibitor solvent DMSO was investigated (Figure 3B,C). The corresponding time-dependent autophosphorylation of GST-tagged CK1δ was determined by measuring phosphate incorporation into GST-CK1δ.

The time-dependent phosphorylation of α-casein, as well as the autophosphorylation of GST-tagged CK1δ, show a typical time-conversion curve starting with a linear region (initial velocity is maximal) and reaching a plateau at later time points. A statistically significant influence of DMSO on phosphorylation of α-casein (Figure 3A) could only be observed for the highest enzyme concentration (335 nM), whereas a statistically significant interaction between the factors time and DMSO was observed for the autophosphorylation level (Figure 3B) at the lowest enzyme concentration (7 nM). More importantly, proportional autophosphorylation of GST-CK1δ showed a statistically significant increase with increasing enzyme concentration (Figure 3C) making up about 40% of the total phosphorylation within reactions containing the highest enzyme concentration (335 nM).

**Figure 4:**
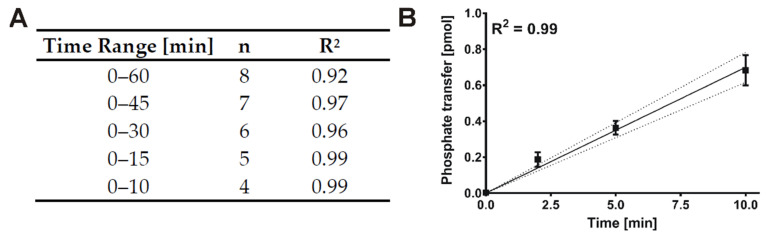
Determination of initial velocity linear region for GST-CK1δ. (**A**) In order to determine the maximum coefficient of determination (R^2^) linear regression analysis was conducted with stepwise decreasing the number of included time points of the product-over-time progression curve determined for GST-CK1δ (7 nM). (**B**) Linear regression for the determined initial velocity region (10 min) including R^2^ value and confidence bands (95%). n, sample size; R^2^, coefficient of determination.

Since SMIs characterized by in vitro kinase reactions are typically dissolved in DMSO, the determination of the initial velocity region was exclusively conducted for kinase reactions containing DMSO to maintain consistency of conditions. Apart from that, the lowest enzyme concentration possible (enzyme concentration vs. signal intensity) was used to minimize the IC_50_ detection limit. According to data presented in Figure 3, a concentration of 7 nM of GST-CK1δ could be used as the signal to background (S/B) ratio obtained with 7 nM concentration was sufficient at each time point measured (S/B ratio ≥ 10). Linear regression analysis with stepwise decreasing the number of included time points of the product-over-time progression curve determined for GST-CK1δ indicated an optimal time range of 10 min (n = 4, R^2^ = 0.99) (Figure 4). According to these results, subsequent determination of K_m (ATP)_ and IC_50_ values using GST-CK1δ was conducted with an enzyme concentration of 7 nM and for a reaction time of 10 min.

### 2.2. Determination of Initial Velocity Region for 6×His-Tagged CK1δ

Similar to GST-tagged CK1δ, the initial velocity region as well as the optimal enzyme concentration were determined for 6×His-tagged CK1δ. Simultaneously, autophosphorylation and the influence of DMSO were also assessed.

**Figure 5:**
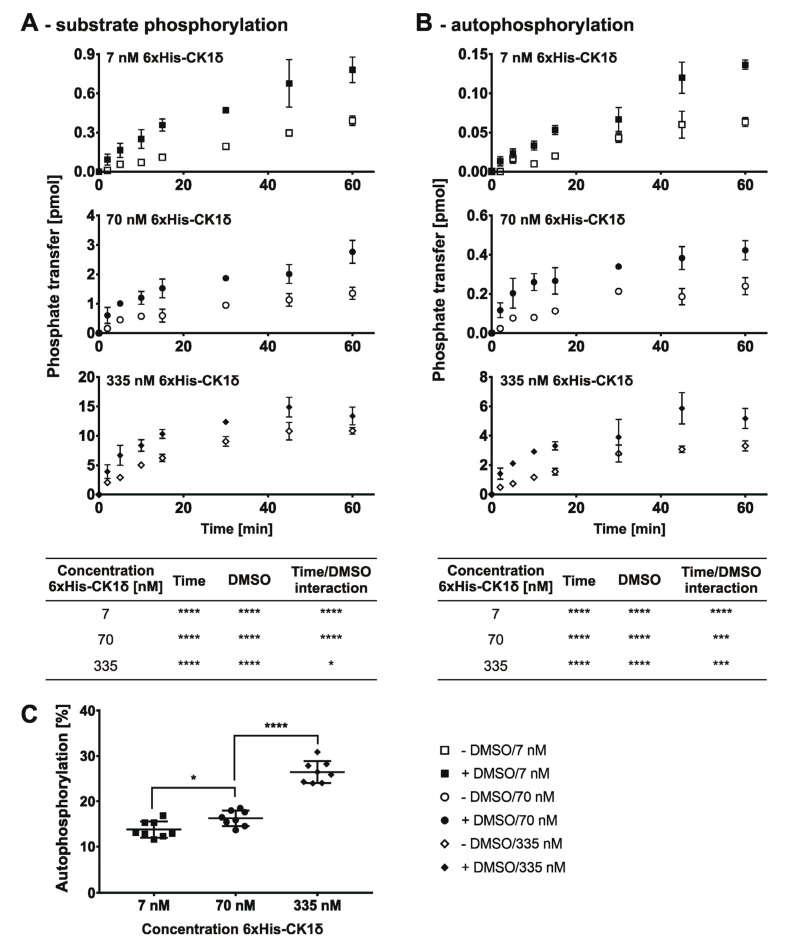
Product-over-time progression curve for 6×His-tagged CK1δ autophosphorylation and substrate phosphorylation. (**A**) Time-dependent phosphate transfer from [γ-^32^P]-ATP to the substrate α-casein catalyzed by human recombinant 6×His-tagged CK1δ using different enzyme concentrations (7, 70, 335 nM) was determined by in vitro kinase reactions in absence and presence of solvent (+/− DMSO). (**B**) Time-dependent autophosphorylation of 6×His-CK1δ has been determined and is presented similar to data shown in (**A**). (**C**) The mean proportional autophosphorylation relative on total phosphorylation (cumulated time-points) is significantly increased at higher enzyme concentrations. Statistical significance was tested using two-way ANOVA (for data presented in (**A**,**B**)) and unpaired two-tailed *t*-test (**C**). * indicates *p* ≤ 0.05, *** indicates *p* ≤ 0.001, **** indicates *p* ≤ 0.0001. DMSO, dimethyl sulfoxide.

**Figure 6:**
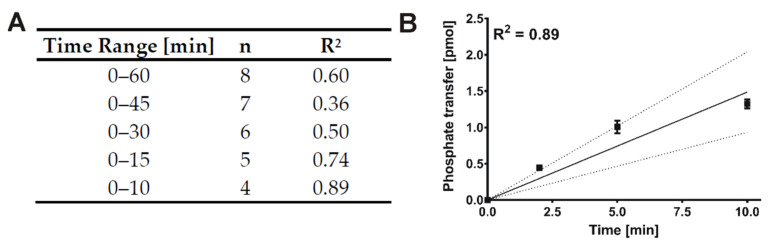
Determination of initial velocity linear region for 6×His-CK1δ. (**A**) In order to determine the maximum coefficient of determination (R^2^) linear regression analysis was conducted with stepwise decreasing the number of included time points of the product-over-time progression curve determined for 6×His-CK1δ (70 nM). (**B**) Linear regression for the determined initial velocity region (10 min) including R^2^ value and confidence bands (95%). n, sample size; R^2^, coefficient of determination.

For the time-dependent phosphorylation of α-casein by 6×His-CK1δ as well as for the time-dependent progression of autophosphorylation, the respective saturation curves could be determined as expected (Figure 5A,B). However, a strong influence of DMSO was observed for all tested enzyme concentrations. Additionally, as previously seen for GST-CK1δ, the proportional autophosphorylation significantly increased with increasing concentrations of 6×His-CK1δ making up about 30% of the total protein phosphorylation within the kinase reactions at the highest enzyme concentration tested (335 nM). Also, for 6×His-CK1δ the lowest enzyme concentration possible (enzyme concentration vs. signal intensity) was used to determine the initial velocity region (Figure 6). In the case of 6×His-CK1δ, a concentration of 70 nM could be used as the signal to background ratio exceeded a value of ten at each time point measured, which was not the case using an enzyme concentration of 7 nM (S/B ratio at 2 min without DMSO = 5.1).

Stepwise decreasing linear regression applied on data of the obtained product-over-time progression curve for 6×His-CK1δ indicated an ideal linear region between 5 and 10 min of reaction time. In order to obtain higher signal, an enzyme concentration of 70 nM and 10 min reaction were used for subsequent experiments to determine the K_m (ATP)_ and IC_50_ values for 6×His-CK1δ.

### 2.3. Determination of K_m_ under Initial Velocity Conditions

**Figure 7:**
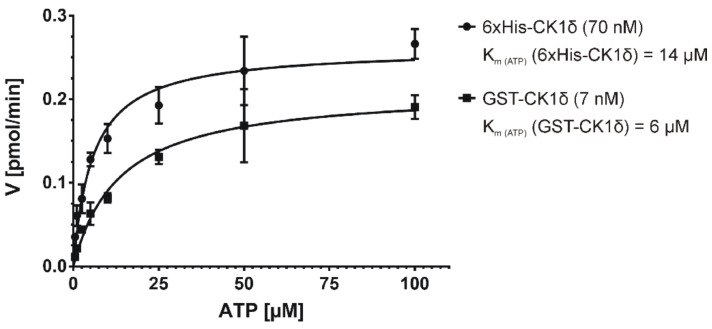
Michaelis-Menten kinetics for GST-CK1δ and 6×His-CK1δ assayed in presence of increasing ATP concentrations. Initial velocity (V_init_) [pmol/min] was determined at different ATP concentrations (0.5 to 100 µM). K_m (ATP)_ defines the concentration of the co-substrate ATP at which half of the maximal velocity (V_max_) is achieved. K_m (ATP)_, Michaelis constant for ATP.

In order to determine the K_m (ATP)_ values for GST-CK1δ and 6×His-CK1δ Michaelis-Menten enzyme kinetic analysis was performed under the defined initial velocity conditions using varying ATP concentrations ranging from 0.5 to 100 µM (Figure 7). The resulting K_m (ATP)_ is defined as the concentration of the co-substrate ATP at which the respective enzymatic velocity of GST-CK1δ or 6×His-CK1δ is half of its maximal value. The calculated K_m (ATP)_ for 6×His-CK1δ was 14 µM, whereas for GST-CK1δ, a K_m (ATP)_ value of 6 µM was determined by using 7 nM concentration of GST-CK1δ and 70 nM of 6×His-CK1δ in kinase reactions performed for 10 min (see data above for determination of ideal/minimal enzyme concentration and initial velocity region). In order to simplify the transformation of the IC_50_ to the K_ic_ value according to the Cheng-Prusoff equation (see introduction section for detailed explanation), ATP concentrations equivalent to the calculated K_m (ATP)_ values were used in subsequent experiments with the aim to determine IC_50_ values.

### 2.4. Determination of IC_50_ Values Using Adapted ATP Concentrations

**Figure 8:**
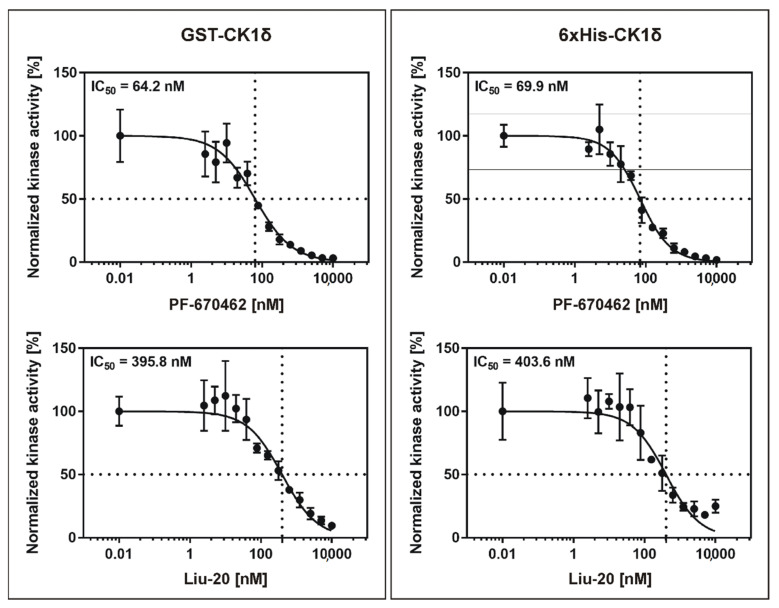
IC_50_ values of inhibitors PF-670462 and Liu-20 for GST-CK1δ and 6×His-CK1δ. In vitro kinase reactions were performed in presence of increasing concentrations (1 to 250 nM) of the CK1-specific inhibitors PF-670462 and Liu-20 using GST-CK1δ (7 nM) or 6×His-CK1δ (70 nM) as kinases. Kinase activity was determined by measuring phosphate incorporation into α-casein and data analysis was performed as described in the materials and methods section. IC_50_, concentration for 50% inhibition.

According to our workflow with the aim of determining comparable parameters for SMIs, IC_50_ values for GST-CK1δ and 6×His-CK1δ were determined using the defined initial velocity conditions and an ATP concentration equivalent to the calculated K_m (ATP)_ values. As appropriate examples for previously reported and established SMIs the CK1-specific ATP-competitive inhibitors PF-670462 [12] and Liu-20 [30] were used for determination of IC_50_ values (Figure 8 and Table 2). The determined IC_50_ values show high comparability between GST- and 6×His-tagged CK1δ: IC_50_ values were calculated with 69.85 nM (PF-670462) and 395.80 nM (Liu-20) for GST-CK1δ and 64.18 nM (PF-670462) and 403.60 nM (Liu-20) for 6×His-CK1δ.

### 2.5. Determination and Comparison of K_ic_ for GST-CK1δ and 6×His-CK1ε Using the CK1ε Specific Inhibitor PF-4800567

In order to prove the concept of the suggested workflow and to demonstrate the superior comparability of K_ic_ values, the inhibitory potential of the CK1ε-specific inhibitor PF-4800567 was determined using GST-tagged CK1δ and 6×His-tagged CK1ε. While the initial velocity region (10 min at 70 nM enzyme concentration) and K_m (ATP)_ (6 µM) for GST-CK1δ have already been defined (see above) these parameters still had to be determined for 6×His-CK1ε.

**Figure 9:**
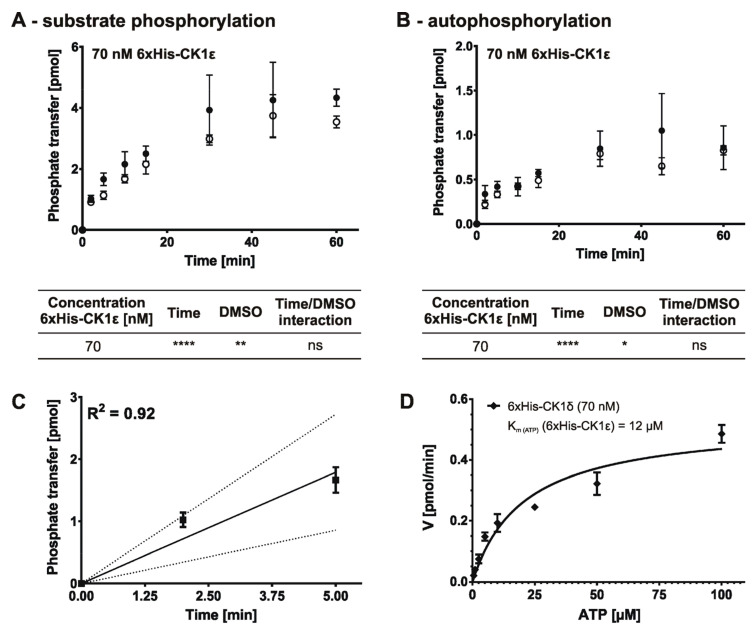
Product-over-time progression curve for 6×His-tagged CK1ε and Michaelis-Menten kinetics for 6×His-CK1ε assayed in presence of increasing ATP concentrations. (**A**) Time-dependent phosphate transfer from [γ-^32^P]-ATP to the substrate α-casein catalyzed by human recombinant 6×His-tagged CK1ε using 70 nM enzyme concentration was determined by in vitro kinase reactions in absence and presence of solvent (+/− DMSO). (**B**) Time-dependent autophosphorylation of 6×His-CK1ε has been determined and is presented similar to data shown in (**A**). (**C**) In order to determine the maximum coefficient of determination (R^2^) linear regression analysis was conducted with stepwise decreasing the number of included time points of the product-over-time progression curve determined for 6×His-CK1ε (70 nM). Linear regression for the determined initial velocity region (5 min) including R^2^ value and confidence bands (95 %) is presented in (**C**). (**D**) Initial velocity (V_init_) [pmol/min] was determined at different ATP concentrations (0.5 to 100 µM). K_m (ATP)_ defines the concentration of the co-substrate ATP at which half of the maximal velocity (V_max_) is achieved. Statistical significance was tested using two-way ANOVA (for data presented in (**A**,**B**). ns indicates *p* > 0.05, * indicates *p* ≤ 0.05, ** indicates *p* ≤ 0.01, **** indicates *p* ≤ 0.0001. DMSO, dimethyl sulfoxide; K_m (ATP)_, Michaelis constant for ATP; n, sample size; R^2^, coefficient of determination.

Because we expected a similar level of activity for 6×His-CK1ε as for the previously tested 6×His-CK1δ, the same enzyme concentration of 70 nM was also used for 6×His-CK1ε. As formerly demonstrated for GST- and 6×His-CK1δ, the solvent DMSO also showed a significant influence on the time-dependent substrate phosphorylation by 6×His-CK1ε (Figure 9A,B). Linear regression analysis with stepwise decreasing the number of included time points indicated an optimal time range of 5 min (R^2^ = 0.92) for the initial velocity region as tested for 70 nM concentration of 6×His-CK1ε (Figure 9C). Consequently, subsequent kinase reactions including the determination of the K_m (ATP)_ and IC_50_ for 6×His-CK1ε were conducted using an enzyme concentration of 70 nM and a reaction time of 5 min.

Michaelis-Menten kinetics for 6×His-CK1ε with varying concentrations of ATP revealed a K_m (ATP)_ value of 21 µM (Figure 9D). Consequently, subsequent determination of IC_50_ values for the CK1ε-specific inhibitor PF-4800567 using GST-CK1δ and 6×His-CK1ε was performed under the defined, enzyme-specific initial velocity conditions with an ATP-concentration equivalent to the calculated K_m_ value (see results presented above for parameters specific for GST-CK1δ).

**Figure 10:**
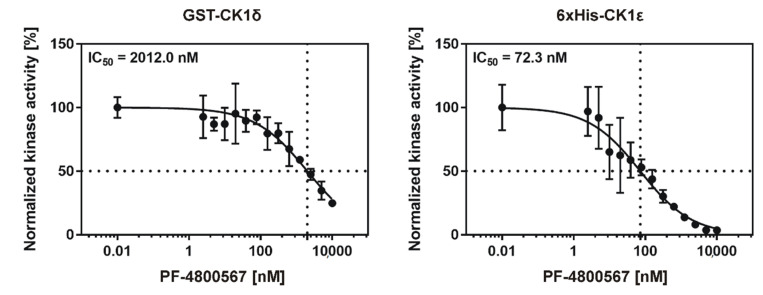
IC_50_ values of inhibitor PF-4800567 for GST-CK1δ and 6×His-CK1ε. In vitro kinase reactions were performed in presence of increasing concentrations (1 to 250 nM) of the CK1ε-specific inhibitor PF-4800567 using GST-CK1δ (7 nM) or 6×His-CK1ε (70 nM) as kinases. Kinase activity was determined by measuring phosphate incorporation into α-casein and data analysis was performed as described in the materials and methods section. IC_50_, concentration for 50% inhibition.

The IC_50_ value using the CK1ε-specific inhibitor PF-4800567 was calculated to be 2012 nM for GST-CK1δ and 72.3 nM for 6×His-CK1ε (Figure 10), thereby confirming the inhibitor’s selectivity for CK1ε. Furthermore, the suggested workflow for determination of highly comparable parameters for SMIs allows the conversion of the calculated IC_50_ values for all tested competitive CK1-specific inhibitors to K_ic_ by implementing Equation (3) as given in the introduction section. By providing K_ic,_ the results will be made highly comparable even among different enzymes. All IC_50_ values, which were determined in the present study along with the resulting K_ic_ values are summarized in Table 2. IC_50_ values previously reported in literature are also included in Table 2. However, it is important to note that these values cannot be easily compared to the values presented here due to significant differences in their experimental and reaction setup (such as luminescent vs. radioactive kinase assay, use of kinase proteins without or with different affinity tags, use of protein or peptide substrates).

## 3. Discussion

In the present study by using the example of CK1, we presented a systematic workflow for the in vitro characterization of SMIs with the aim to achieve more universal comparability, even between different tested enzymes. Thorough determination of the initial velocity region as well as defining an ATP concentration equal to K_m (ATP)_ enables the conversion of the obtained IC_50_ values to the inhibitor constant K_i_, an enzyme-specific variable that is independent of the experimental setup and universally comparable.

In general, certain considerations need to be made before setting up screening approaches to determine kinase activity and inhibitory potency of SMIs. As mentioned earlier, variability in protein kinase purification approaches (GST vs. 6×His affinity tag) or the effects of intramolecular autophosphorylation need to be taken into account. While performing this study, we showed that autophosphorylation of CK1δ is significantly increased at higher enzyme concentrations. Consequently, problems may arise using luci-ferase-based kinase assays, which measure consumption of the co-substrate ATP or the generation of ADP in general, and thus do not differentiate between phosphorylation events associated with substrate phosphorylation or autophosphorylation [15,16]. With the enzyme concentrations used in this study, substrate phosphorylation is overestimated with an error of 36% (335 nM GST-CK1δ) or 26% (335 nM 6×His-CK1δ, see data presented in Figure 3 and Figure 5) when phosphorylation is not differentiated and autophosphorylation is included in measurement of total phosphorylation events. Therefore, we propose to use kinase assay setups, like the herein described radioactive based assay, allowing distinctions between kinase autophosphorylation and substrate phosphorylation to minimize false positive signals. By separating kinase reactions in SDS-PAGE and subjecting excised protein bands to Cherenkov counting, substrate phosphorylation and autophosphorylation can be clearly distinguished. However, we are aware of the limitations of the in vitro kinase assay using radiolabeled [γ-^32^P]-ATP such as exposure to radioactivity, the elaborate disposal of radioactive waste and the use of protein substrates instead of easy to use peptide substrates. Nevertheless, the high sensitivity and significant signal-to-noise ratio of the radioactive based method enables the use of low enzyme concentrations. This is indispensable for the investigation of highly effective inhibitors as the kinase concentration defines the lower limit of detectable IC_50_ value by half of the used enzyme concentration (assumed that the inhibitor binds to the kinase proteins in a one-to-one ratio) [31]. However, the lowest enzyme concentration possible also needs to be selected based on a beneficial signal-to-noise ratio and only enzyme concentrations resulting in clearly detectable phosphorylation signals should be chosen for subsequent analysis.

In addition, we observed that the presence of DMSO significantly affected the measured enzyme activity up to 50%, as observed for 6×His-CK1δ. This influence of DMSO on enzyme activity showed that consideration of the experimental setup (solvents, buffers, pH) is pivotal for enzyme kinetics and should be kept constant throughout the experimental workflow, otherwise it is likely to falsifying the results. One should point out, that most organic compounds like SMIs are dissolved in the organic solvent DMSO, which has to be considered in the assay composition even in the initial experiments to eradicate this potential source of error [32]. Although DMSO still is generally considered an irreplaceable solvent, it might also be worthwhile to consider alternatives like zwitterion liquid (ZIL), which recently has been characterized as multifunctional and biocompatible solvent compensating many shortcomings noticed for DMSO [33].

The first part of the proposed workflow aims to determine the initial velocity region of the enzyme reaction as well as the optimal enzyme concentration. As a basic principle, one should realize that the lower detection limit of the IC_50_ to be determined is restricted by the used enzyme concentration. When testing highly potent inhibitors, the lowest possible enzyme concentration should be used, still maintaining a proper signal-to-noise ratio. Although depletion of ATP catalyzed by all tested enzymes was less than 10% of total ATP after 60 min, the reaction velocity already decreased after 10 min (as shown for 7 nM GST-CK1δ and 70 nM 6×His-CK1δ in Figure 3 and Figure 5) or 5 min (as shown for 70 nM 6×His-CK1ε in Figure 9). Since substrate depletion cannot explain the decline in reaction velocity, CK1δ might be inhibited due to time-dependent increase in autophosphorylation [18,34,35]. Unfortunately, autophosphorylation events within the enzyme reaction cannot be prevented in an easy way, e.g., by the addition of phosphatases.

As a final step in our in vitro kinase assay workflow, we determined the IC_50_ values of the published compounds PF-670462 and Liu-20 using two different recombinantly expressed and purified enzymes, GST-CK1δ and 6×His-CK1δ. Under the conditions, which were determined in previous experiments (initial velocity region, optimal enzyme concentration, ATP concentration at K_m (ATP)_), the IC_50_ determination for the selected inhibitors showed similar values for both differently purified and affinity-tagged CK1δ kinases. In our case, the influence of different affinity tags was negligible, as can be seen from the highly similar IC_50_ and K_ic_ values determined for GST-CK1δ and 6×His-CK1δ (see Table 1). However, the effects of used affinity-tags might have to be determined in a kinase- or experiment-specific fashion.

When comparing the IC_50_ data provided in Table 2, it has to be considered that, as expected, our calculated IC_50_ values for PF-670462, Liu-20 and PF-4800657 differ from the previously published IC_50_ values of 13 nM for PF-670462 and CK1δ [13], 86 nM for Liu-20 [30], 711 nM for PF-4800567 and CK1δ as well as 32 nM for PF-4800567 and CK1ε [13] (see Table 2). Apart from the possible occurrence of batch-to-batch variability for the tested SMIs (which is beyond the scope of this manuscript and will not be discussed further), these differences might arise because the initial velocity region for performing in vitro kinase reactions has not been determined properly, or a differentiation between substrate phosphorylation and autophosphorylation has not been possible due to assay design and the analysis of total phosphorylation events, as this was the case for the previous determination of IC_50_ values for PF-670462 and PF-4800567 [13]. For determining these values, a luminescence-based assay setup detecting remaining ATP levels in the kinase reactions was used [13]. Furthermore, discrepancies in IC_50_ values might also arise from different ATP concentrations used in different experimental setups. While this was not the case for the original data reported for Liu-20, PF-670462 and FP-4800567 (all tested at 10 µM concentration [13,30]), this is a common problem observed in literature (reviewed for CK1-specific inhibitors in [29]). Instead of using arbitrary ATP concentrations for in vitro kinase reactions, inhibitors should be tested at ATP concentrations equal to K_m (ATP)_ for the enzyme to be tested, as suggested by our workflow. Finally, in order to ensure comparability of the parameters describing inhibitor potency between different inhibitors and enzymes, the K_i_ value should be determined and stated by default. In our presented example experiments and calculations, only ATP-competitive CK1-specific inhibitor compounds were used, for which Equation (3) for competitive inhibitor binding can easily be implemented to determine K_ic_. However, while concentration-specific effects of kinase, substrate and ATP are already considered when calculating K_i_ or can be neglected, the types of kinase protein (e.g., clear designation of isoforms and splice variants or the use of truncated proteins) and substrate to be phosphorylated need to be provided when describing the performed kinase reactions. In the case of CK1δ, it has to be considered that the C-termini of the various splice variants differ in length, sequence and their site-specific phosphorylation pattern [28]. Due to its influence on kinase conformation and activity this difference also influences IC_50_ and K_i_ values [36,37].

## 4. Material and Methods

### 4.1. Expression and Purification of GST-Tagged CK1δ

Expression of recombinant GST-human CK1δ (transcription variant 1; NM_001893 [38]) was induced by adding 1 mM IPTG to diluted overnight bacteria cultures at an OD600 of 0.7 to 0.9 AU. After protein overexpression for 18 h at 18 °C bacteria cultures were harvested by centrifugation. Bacteria pellets were stored at −80 °C until further use. Bacteria cell lysis was performed by using lysozyme in *GST* lysis buffer (20 mM Tris-HCl (pH 7.6), 150 mM NaCl, 10% [*v*/*v*] glycerol, 0.5% [*v*/*v*] NP40, 1 mM EDTA, 1 mM EGTA, 1 mM benzamidine, 1 mM aprotinin, 1 mM DTT) on ice for 30 min. Bacterial DNA was fragmented via ultrasonic sound treatment (Thermo Fisher Scientific Inc., Waltham, MA, USA). The lysate was cleared by centrifugation at 10,000 rpm and 4 °C for 30 min. Using an automated FPLC system (EttanLC, GE Healthcare, Chalfont St Giles, GB) the filtered supernatant was loaded onto an equilibrated GSTrap FF 1 mL column (Cytiva, Freiburg, Germany) at a flow rate of 0.5 mL/min. Column was washed with lysis buffer until UV detection at 280 nm reached a stable baseline. Column-bound protein was eluted stepwise using elution buffer (50 mM Tris-HCl (pH 7.6), 5 mM reduced glutathione, 1 mM EDTA) and eluted protein was finally dialyzed against glutathione-free elution buffer three times for 10 min each.

### 4.2. Expression and Purification of 6×His-Tagged CK1δ and 6×His-Tagged CK1ε

Expression and harvest of bacterial cultures of recombinant 6×His-human CK1δ (transcription variant 1; NM_001893 [38]) and 6×His-human CK1ε (transcription variant 1; NM_152221 [39]) were performed as indicated above for GST-CK1δ. Bacteria cell lysis was performed as mentioned above by using 6×His lysis buffer, which is composed of 20 mM Tris-HCl (pH 7.6), 150 mM NaCl; 10 % [*v*/*v*] glycerol, 1 mM benzamidine and 1 mM aprotinin. Cleared and filtered lysate was loaded onto cOmplete^TM^ His-Tag purification 1 mL column (Roche, Mannheim, Germany) at a flow rate of 0.5 mL/min. The column was washed with a gradient from lysis buffer to of washing buffer (20 mM Tris-HCl (pH 7.6), 50 mM NaCl, 10% [*v*/*v*] glycerol, 1 mM aprotinin) at 0.5 mL/min within 30 min. Afterwards, proteins were eluted with a gradient from washing buffer to 6×His elution buffer (20 mM Tris-HCl (pH 7.6), 250 mM imidazole) within 3 min. Eluted protein was dialyzed against imidazole-free elution buffer three times for 10 min each.

### 4.3. In Vitro Kinase Reactions

All in vitro kinase reactions were performed at 30 °C in a total volume of 15 µL containing 25 mM Tris-HCl (pH 7.0), 10 mM MgCl_2_, 100 µM EDTA and 0.4 pmol [γ-^32^P]-ATP. Substrate (α-casein) was used at 2.5 (initial experiments) or 10 µM (IC_50_ determination) and ATP concentrations were used within a range from 0.5 to 100 µM, respectively, depending on the experimental setup. GST- or 6×His-tagged CK1δ or CK1ε were used as enzymes in 7, 70 or 335 nM concentration. Additionally, a reaction without enzyme was performed and used to determine the background signal specifying the minimal usable enzyme concentration (signal to background ratio ≥10). Reactions were stopped by adding 3 µL of 5× SDS loading buffer and boiling at 95 °C for 5 min. Proteins were separated by SDS-PAGE and stained with Coomassie Brilliant Blue R250 (Waldeck GmbH & Co. KG, Muenster, Germany). Radioactively labelled proteins were visualized by autoradiography. For quantification, phosphorylated proteins were excised from dried gels and phosphate incorporation was measured via Cherenkov counting.

For the determination of V_max_ and K_m_, various ATP concentrations in a range from 0.5 to 100 µM were tested. Raw data was used to calculate enzyme velocity (V) (phosphate transfer per min). V was plotted over ATP concentration and fitted to the Michaelis-Menten model using GraphPad Prism 7 (GraphPad Software, La Jolla, CA, USA). K_m_ was calculated as the concentration of substrate needed to get half-maximal velocity. In order to determine IC_50_ values, raw data was transformed from initially measured cpm values to pmol of transferred phosphate. Subsequently data was transformed logarithmically (X = log(X)), normalized to DMSO (100%) and zero (0%) and finally fit to sigmoidal dose-response curves (variable slope) with nonlinear regression using GraphPad Prism 7.

### 4.4. Statistical Analysis

All measurements were carried out as triplets (n = 3). Outliers based on technical errors were removed and excluded from statistical analysis by visual inspection of data. Comparison of the product-over-progression curves was performed with a two way ANOVA (GraphPad Prism 7) to determine the influence of the factors time and DMSO, as well as their interaction. Comparison between proportional autophosphorylation was performed with an unpaired two-tailed *t*-test (α = 0.05, homogeneity of variances) or with an unpaired two-tailed *t*-test with Welch’s correction (α = 0.05, heterogeneity of variances) after normal distribution was confirmed (Shapiro-Wilk).

## 5. Conclusions

The increasing importance of protein kinases in several pathological conditions led to the development of highly active SMIs with the intention to find novel ways of treatment. The continuous improvement of lead structures is one of the key steps in the development process of SMIs and is based on the results gained from in vitro kinase assays. However, a lack of comparability due to interlaboratory variation evoked by unstandardized methods and the implementation of high-throughput screenings, based on different assay formats strongly affects the progress made in this field of research. Herein we provided a workflow for verification and validation of high-throughput screening data as well as reduction of interlaboratory variation.

The main and basic step of this workflow is the initial characterization of the enzyme used in the assay by determination of the optimal enzyme concentration combined with the definition of the initial velocity region. Further, after determining the K_m_ value for ATP the IC_50_ value of the inhibitors to be tested can be determined using the previously defined conditions. As a proof of principle by following this procedure, we were able to generate highly comparable K_i_ values using two SMIs (PF-670462 and Liu-20) and two different tagged variants of CK1δ (GST vs. 6×His) from independent batches and purification procedures mimicking two different working procedures from two different laboratories. Furthermore, we demonstrate that enzyme-specific properties like autophosphorylation or reaction-specific conditions like solvents (DMSO) can strongly falsify the results of the assay. Therefore, understanding and evaluating the impact of these variables on the enzyme reaction is a mandatory element in the procedure of standardization and should not be neglected.

Although the assay conditions described in the present study were optimized for CK1, the proposed systematic workflow can easily be established for any other enzymes or substrates independently of the assay design. The implementation of a standardized workflow for kinase reactions and inhibitor characterization allowing the presentation of results with the aid of K_i_ instead of an IC_50_ value highly facilitates the comparability and ranking of different SMIs, thereby providing superior efficiency for the in-silico modeling and subsequent in vitro characterization of new inhibitor compounds.

## Figures and Tables

**Table 1:** Interlab variation of IC_50_ values for CK1-specific inhibitors.

	Working Group A				Working Group B			
Inhibitor	IC_50_ [µM]	ATP [µM]	Isoform	Ref.	IC_50_ [µM]	ATP [µM]	Isoform	Ref.
IC261	1	10	CK1δ	[9]	2.5	100	CK1δ **	[10]
D4476	0.3	20	CK1δ *	[11]	0.2	100	CK1δ **	[10]
PF-670462	0.008	5	CK1ε *	[12]	0.08	10	CK1ε	[13]

* Use of C-terminally truncated kinases (CK1δ(1–294) and CK1εΔ319, respectively). ** Use of CK1δ of yeast origin (*Saccharomyces pombe*). IC_50_, concentration for 50% inhibition.

**Table 2:** Overview of the tested (**A**) enzymes and (**B**) inhibitors as well as the determined IC_50_ values and the resulting K_ic_ values.

(**A**)				
**Enzyme**	**Inhibitor**	**IC_50_ [nM]**	**K_ic_ [nM]**	**IC_50_ in Literature [nM]**
GST-CK1δ	PF-670462	69.85	34.93	13 * [13]
	Liu-20	395.80	197.90	86 [30]
	PF-4800567	2012.00	1006.00	711 * [13]
6×His-CK1δ	PF-670462	64.18	32.09	-
	Liu-20	403.60	201.80	-
6×His-CK1ε	PF-4800567	72.30	36.15	32 ** [13]
(**B**)				
**Inhibitor**	**PF-670462**	**PF-4800567**		**Liu-20**
Structure	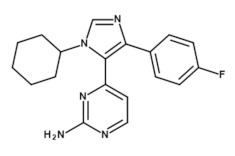	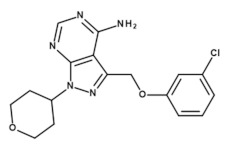		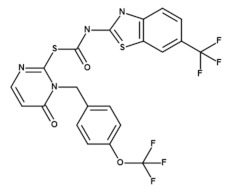
Reference	[12]	[13]		[30]

* IC_50_ values were determined using untagged CK1δ and a CK1-specific peptide substrate. ** IC_50_ value was determined using 6×His-CK1ε and a CK1-specific peptide substrate. IC_50_, concentration for 50% inhibition; K_ic_, inhibitor constant (competitive inhibitor).

## Data Availability

The data presented in this study are available in the article and on request from the corresponding author.

## Sample Availability

Samples of the compounds PF-670462 and PF-4800567 are commercially available. Samples of the compound Liu-20 are available from the authors.

## References

[1] Ardito F., Giuliani M., Perrone D., Troiano G., Lo Muzio L. (2017). The crucial role of protein phosphorylation in cell signaling and its use as targeted therapy (Review). Int. J. Mol. Med..

[2] Varedi K.S.M., Ventura A.C., Merajver S.D., Lin X.N. (2010). Multisite phosphorylation provides an effective and flexible mechanism for switch-like protein degradation. PLoS ONE.

[3] Cheng H.-C., Qi R.Z., Paudel H., Zhu H.-J. (2011). Regulation and function of protein kinases and phosphatases. Enzyme Res..

[4] Sridhar R., Hanson-Painton O., Cooper D.R. (2000). Protein kinases as therapeutic targets. Pharm. Res..

[5] Benn C.L., Dawson L.A. (2020). Clinically Precedented Protein Kinases: Rationale for Their Use in Neurodegenerative Disease. Front. Aging Neurosci..

[6] Fabbro D., Cowan-Jacob S.W., Moebitz H. (2015). Ten things you should know about protein kinases: IUPHAR Review 14. Br. J. Pharmacol..

[7] Miljković F., Bajorath J. (2018). Computational Analysis of Kinase Inhibitors Identifies Promiscuity Cliffs across the Human Kinome. ACS Omega.

[8] Cohen P., Cross D., Jänne P.A. (2021). Kinase drug discovery 20 years after imatinib: Progress and future directions. Nat. Rev. Drug Discov..

[9] Mashhoon N., DeMaggio A.J., Tereshko V., Bergmeier S.C., Egli M., Hoekstra M.F., Kuret J. (2000). Crystal structure of a conformation-selective casein kinase-1 inhibitor. J. Biol. Chem..

[10] Rena G., Bain J., Elliott M., Cohen P. (2004). D4476, a cell-permeant inhibitor of CK1, suppresses the site-specific phosphorylation and nuclear exclusion of FOXO1a. EMBO Rep..

[11] Bain J., Plater L., Elliott M., Shpiro N., Hastie C.J., McLauchlan H., Klevernic I., Arthur J.S.C., Alessi D.R., Cohen P. (2007). The selectivity of protein kinase inhibitors: A further update. Biochem. J..

[12] Badura L., Swanson T., Adamowicz W., Adams J., Cianfrogna J., Fisher K., Holland J., Kleiman R., Nelson F., Reynolds L. (2007). An inhibitor of casein kinase I epsilon induces phase delays in circadian rhythms under free-running and entrained conditions. J. Pharmacol. Exp. Ther..

[13] Walton K.M., Fisher K., Rubitski D., Marconi M., Meng Q.-J., Sládek M., Adams J., Bass M., Chandrasekaran R., Butler T. (2009). Selective inhibition of casein kinase 1 epsilon minimally alters circadian clock period. J. Pharmacol. Exp. Ther..

[14] Myers S.M., Bawn R.H., Bisset L.C., Blackburn T.J., Cottyn B., Molyneux L., Wong A.-C., Cano C., Clegg W., Harrington R.W. (2016). High-Throughput Screening and Hit Validation of Extracellular-Related Kinase 5 (ERK5) Inhibitors. ACS Comb. Sci..

[15] Kashem M.A., Nelson R.M., Yingling J.D., Pullen S.S., Prokopowicz A.S., Jones J.W., Wolak J.P., Rogers G.R., Morelock M.M., Snow R.J. (2007). Three mechanistically distinct kinase assays compared: Measurement of intrinsic ATPase activity identified the most comprehensive set of ITK inhibitors. J. Biomol. Screen..

[16] Koresawa M., Okabe T. (2004). High-throughput screening with quantitation of ATP consumption: A universal non-radioisotope, homogeneous assay for protein kinase. Assay Drug Dev. Technol..

[17] Romano P.R., Garcia-Barrio M.T., Zhang X., Wang Q., Taylor D.R., Zhang F., Herring C., Mathews M.B., Qin J., Hinnebusch A.G. (1998). Autophosphorylation in the activation loop is required for full kinase activity in vivo of human and yeast eukaryotic initiation factor 2alpha kinases PKR and GCN2. Mol. Cell. Biol..

[18] Cullati S., Chen J.-S., Gould K. (2020). Autophosphorylation of the CK1 kinase domain regulates enzyme activity and function. FASEB J..

[19] Beenstock J., Mooshayef N., Engelberg D. (2016). How Do Protein Kinases Take a Selfie (Autophosphorylate)?. Trends Biochem. Sci..

[20] Bhoir S., Shaik A., Thiruvenkatam V., Kirubakaran S. (2018). High yield bacterial expression, purification and characterisation of bioactive Human Tousled-like Kinase 1B involved in cancer. Sci. Rep..

[21] Akizuki K., Toyama T., Yamashita M., Sugiyama Y., Ishida A., Kameshita I., Sueyoshi N. (2018). Facile preparation of highly active casein kinase 1 using Escherichia coli constitutively expressing lambda phosphatase. Anal. Biochem..

[22] Gietzen K.F., Virshup D.M. (1999). Identification of inhibitory autophosphorylation sites in casein kinase I epsilon. J. Biol. Chem..

[23] Budini M., Jacob G., Jedlicki A., Pérez C., Allende C.C., Allende J.E. (2009). Autophosphorylation of carboxy-terminal residues inhibits the activity of protein kinase CK1alpha. J. Cell. Biochem..

[24] Brooks H.B., Geeganage S., Kahl S.D., Montrose C., Sittampalam S., Smith M.C., Weidner J.R., Markossian S., Sittampalam G.S., Grossman A., Brimacombe K., Arkin M., Auld D., Austin C.P., Baell J., Caaveiro J.M., Chung T.D. (2004). Basics of Enzymatic Assays for HTS. Assay Guidance Manual.

[25] Wang Y., Guan J., Di Trani J.M., Auclair K., Mittermaier A.K. (2019). Inhibition and Activation of Kinases by Reaction Products: A Reporter-Free Assay. Anal. Chem..

[26] Knight Z.A., Shokat K.M. (2005). Features of selective kinase inhibitors. Chem. Biol..

[27] Knippschild U., Gocht A., Wolff S., Huber N., Löhler J., Stöter M. (2005). The casein kinase 1 family: Participation in multiple cellular processes in eukaryotes. Cell. Signal..

[28] Xu P., Ianes C., Gärtner F., Liu C., Burster T., Bakulev V., Rachidi N., Knippschild U., Bischof J. (2019). Structure, regulation, and (patho-)physiological functions of the stress-induced protein kinase CK1 delta (CSNK1D). Gene.

[29] Knippschild U., Krüger M., Richter J., Xu P., García-Reyes B., Peifer C., Halekotte J., Bakulev V., Bischof J. (2014). The CK1 Family: Contribution to Cellular Stress Response and Its Role in Carcinogenesis. Front. Oncol..

[30] Liu C., Witt L., Ianes C., Bischof J., Bammert M.-T., Baier J., Kirschner S., Henne-Bruns D., Xu P., Kornmann M. (2019). Newly Developed CK1-Specific Inhibitors Show Specifically Stronger Effects on CK1 Mutants and Colon Cancer Cell Lines. Int. J. Mol. Sci..

[31] Goldstein A. (1944). The mechanism of enzyme-inhibitor-substrate reactions: Illustrated by the cholinesterase-physostigmine-acetylcholine system. J. Gen. Physiol..

[32] Wolf A., Shimamura S., Reinhard F.B.M. (2012). Working with small molecules: Preparing and storing stock solutions and determination of kinetic solubility. Methods Mol. Biol..

[33] Kuroda K., Komori T., Ishibashi K., Uto T., Kobayashi I., Kadokawa R., Kato Y., Ninomiya K., Takahashi K., Hirata E. (2020). Non-aqueous, zwitterionic solvent as an alternative for dimethyl sulfoxide in the life sciences. Commun. Chem..

[34] Rivers A., Gietzen K.F., Vielhaber E., Virshup D.M. (1998). Regulation of casein kinase I epsilon and casein kinase I delta by an in vivo futile phosphorylation cycle. J. Biol. Chem..

[35] Graves P.R., Roach P.J. (1995). Role of COOH-terminal phosphorylation in the regulation of casein kinase I delta. J. Biol. Chem..

[36] Bischof J., Leban J., Zaja M., Grothey A., Radunsky B., Othersen O., Strobl S., Vitt D., Knippschild U. (2012). 2-Benzamido-N-(1H-benzo[d]imidazol-2-yl)thiazole-4-carboxamide derivatives as potent inhibitors of CK1δ/ε. Amino Acids.

[37] Richter J., Bischof J., Zaja M., Kohlhof H., Othersen O., Vitt D., Alscher V., Pospiech I., García-Reyes B., Berg S. (2014). Difluoro-dioxolo-benzoimidazol-benzamides As Potent Inhibitors of CK1δ and ε with Nanomolar Inhibitory Activity on Cancer Cell Proliferation. J. Med. Chem..

[38] Kusuda J., Hidari N., Hirai M., Hashimoto K. (1996). Sequence analysis of the cDNA for the human casein kinase I delta (CSNK1D) gene and its chromosomal localization. Genomics.

[39] Fish K.J., Cegielska A., Getman M.E., Landes G.M., Virshup D.M. (1995). Isolation and characterization of human casein kinase I epsilon (CKI), a novel member of the CKI gene family. J. Biol. Chem..

